# Supplementary material for: Anterior cingulate neurons combine outcome monitoring of past decisions with ongoing movement signals
Source: Nat Commun. 2026 Mar 24;17:4354. doi: 10.1038/s41467-026-70639-1 (PMC13171896; doi:10.1038/s41467-026-70639-1)
Supplement: Supplementary file 1 — Supplementary Information [file 41467_2026_70639_MOESM1_ESM.pdf]

Supplementary Information for:

Anterior cingulate neurons combine outcome monitoring of past decisions with ongoing movement signals

Lukas T. Oesch, Makenna C. Thomas, Davis Sandberg, João Couto and Anne K. Churchland

This document contains:

Supplementary Figures 1 – 5

Supplementary Tables 1 & 2

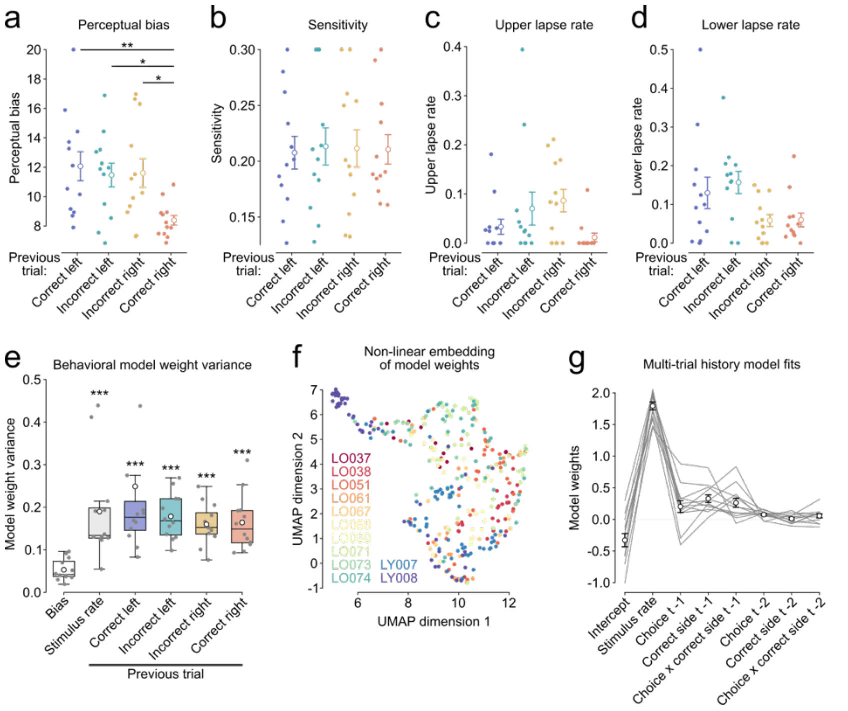

Supplementary figure 1. The behavioral influence of most recent trial history varies between sessions. *(a, b, c, d) Estimates of the psychometric parameters, bias (a), sensitivity (b), upper lapse rate (c), and lower lapse rate (d). Scatter points on the left represent individual subjects while dots with errorbars depict mean ± sem over subjects. Linear mixed-effects models with random effect of subject. Coefficient test were two-tailed and P-values are adjusted for multiple comparisons. *P < 0.05, **P < 0.01. (e) Variance of the session-by-session weights of the behavioral choice decoding. Boxes represent median ± interquartile range, whiskers are 1.5x the interquartile range, and dots depict the mean. Linear mixed-effects model with random effect of subject. All comparisions are two-tailed and are made agains the bias (intercept). ***P < 0.001. (f) Non-linear embedding of the behavioral choice decoding weights. Scatter points represent sessions color-coded by subject. (g) Behavioral decoding models including regressors for most recent trial history (t-1) and trial history from two trials back (t-2). Lines are individual subjects while dots with errorbars show mean ±sem over subjects.*

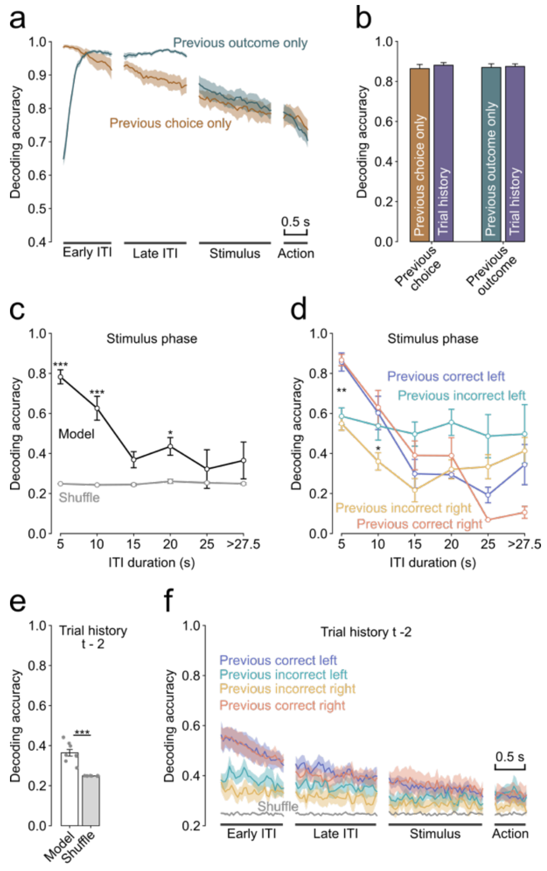

Supplementary Figure 2. Decoders trained on trial history decode previous choice and outcome equally well as corresponding binary classifiers. *(a) Mean ± sem. decoding accuracy across trial time for decoders trained on previous choice- (orange) or previous outcome (teal) only. (b) Mean ± sem neural decoding accuracy for previous choice or previous outcome for their corresponding binary decoders or trial history decoders (trained to decode combinations of previous choices and outcomes), linear mixed-effects model with session nested in subject as random effects. No significant effect of model type, decoded variable or their interaction was found (two-tailed test on model coefficients). (c) Average ± sem trial history decoding accuracy during the stimulus phase for different preceding ITI durations. The ITI duration is defined here as the time between outcome delivery in the preceding trial and current trial initiation (see also Figure 1b). ***P < 0.001, *P < 0.05, linear mixed-effects model with sessions nested in subjects as random effects, two tailed tests on model coefficients. (d) Mean ± sem trial history decoding accuracy during the stimulus phase for different preceding ITI durations split by trial history context. Asterisks mark significant differences between the previous correct and previous incorrect trial history contexts for the 5 s bin and between either of the previous correct versus the previous incorrect right contexts for the 10 s bin. **P < 0.01, *P < 0.05, linear mixed-effects model with sessions nested in subjects as random effects. Coefficient testing was two-tailed, and P-values were adjusted for multiple comparisons. (e) Average ± sem decoding accuracy across all timepoints for trial events occurring two (rather than only one) trial back, ***P < 0.001, linear mixed-effects model with sessions nested in subjects as random effects, two-tailed test on model coefficients. (f) Mean ± sem decoder accuracy over trial time for different trial history contexts.*

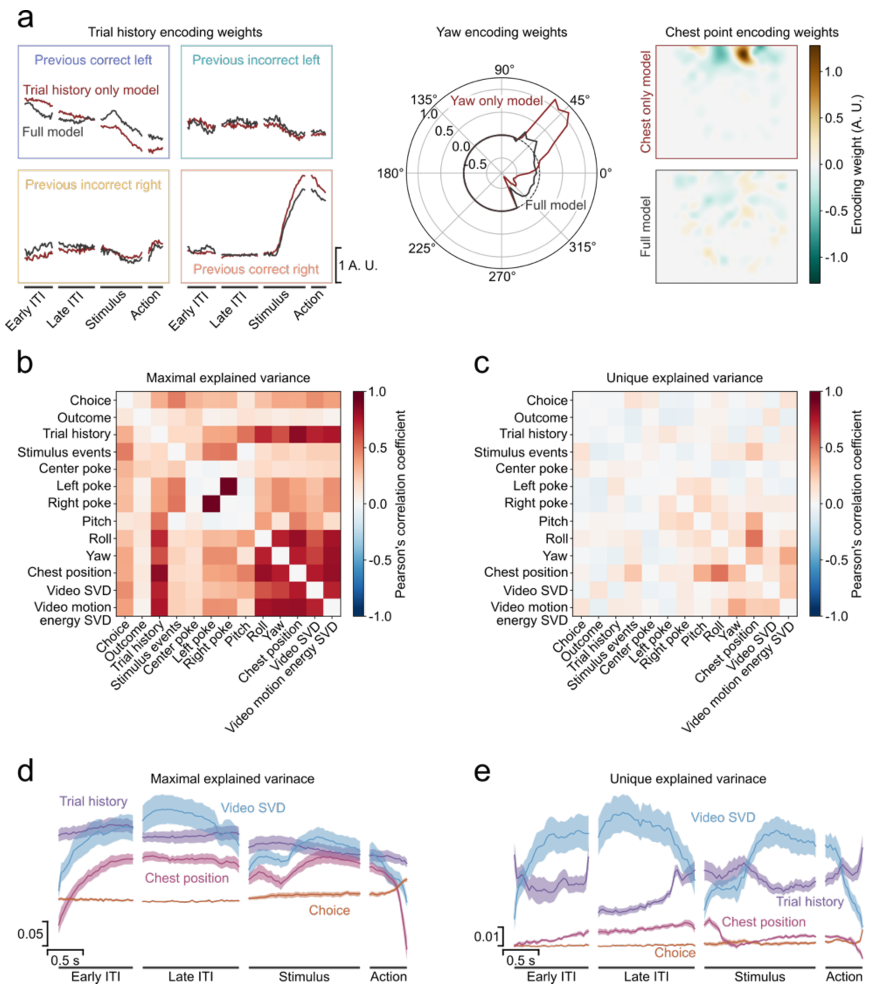

Supplementary Figure 3. The linear encoding model identifies the unique contribution of regressors to the neural activity. *(a) Comparison of encoding model weights from an example neuron for model where the regressors for all except a single variable are shuffled (red) and the full models without shuffling (dark grey). Trial history weights (left), yaw tuning (center) and chest point tuning (right) are shown. (b) Correlation between maximal explained variance for different variables in all neurons from an example session. Note the high correlation between trial history and yaw and chest point. (c) Correlation of the unique explained variance between variables for the same session as in (b). (d and e) Mean ± sem reconstructed time course of maximal (d) and unique explained variance (e) for different variables.*

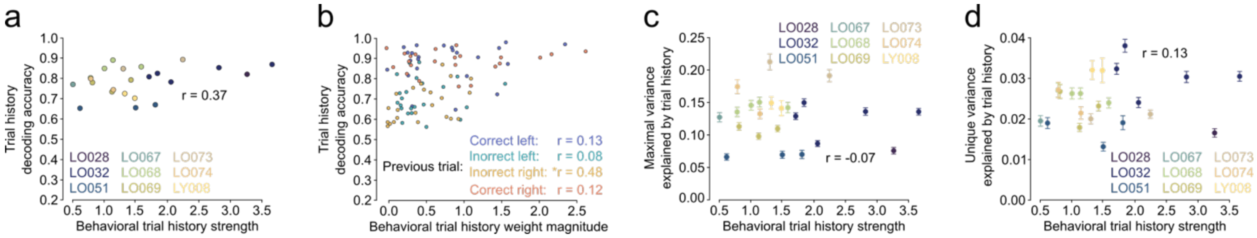

Supplementary figure 4. Neural encoding of trial history in ACC is not correlated with the strength of trial history biases. *(a) Neural population decoding of trial history is not correlated with the behavioral usage of trial history information. Each point is a single animal. Person's correlation coefficient with two-tailed permutation test. (b) Decoding accuracy for specific trial history contexts is independent of the magnitude of the corresponding behavioral model weight. *P < 0.05, Person's correlation coefficient with two-tailed permutation test. Note that individual P-values are not corrected for multiple comparisons. (c and d) Neither maximal nor unique variance of individual neurons explained by trial history are correlated with behavioral trial history biases. Dots and error bars represent the mean ±sem over neurons for each session, r indicates the Person's correlation coefficient with two-tailed permutation test.*

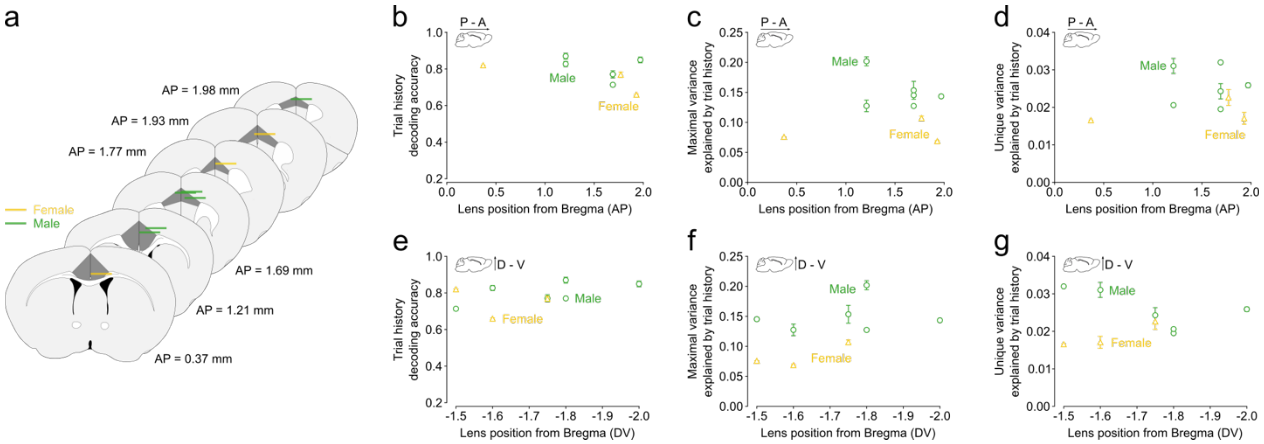

Supplementary Figure 5: Female mice show weaker trial history representations than male mice. *(a) Anatomical reconstruction of approximate GRIN lens position (its center) separated by subject sex. Coronal brain section schematics are adapted from[75]. (b and e) Mean ± sem trial history decoding accuracy by lens position along the anterior-posterior (b) and dorsal-ventral axis (e) separated by subject sex. We found a significant main effect of sex ($P < 0.05$) but no effects for AP or DV lens position or their interaction, linear-mixed effects model with subject as random effect, two-tailed test on model coefficients. (c and f) Mean ± sem maximal explained variance for trial history as a function of GRIN lens position and color-coded by sex. We observed significant effects of sex ($P < 0.01$), AP position ($P < 0.01$), DV position ($P < 0.01$) and the interaction between AP and DV position ($P < 0.01$), linear mixed effects model with random effect for subject and two-tailed test on coefficients. (d and g) Average± sem unique explained variance for trial history by GRIN lens AP (d) and DV (g) position separated by subject sex. We found significant effects of sex ($P < 0.01$), but no effects for AP or DV position or their interaction, linear mixed-effects model with subject as random effect, two-tailed test on model coefficients. Sagittal brain sections on panels b – g were adapted from[75].*

Subject ID	Sex	Stimulus modality	Number of sessions included In Figure 1	Number of sessions with full stimulus set	Average performance on easiest trials	Average number of valid trials	Average early withdrawal rate	Average number of no-choice trials	Average trial history strength
LO028	F	Auditory	-	-	-		-	-	-
LO032	M	Auditory	-	-	-		-	-	-
LO037	M	Visual	12	0	0.790	189	0.322	0.000	1.055
LO038	M	Visual	26	3	0.830	152	0.389	0.000	0.970
LO051	F	Visual	40	25	0.832	180	0.290	0.100	0.976
LO061	F	Visual	18	17	0.801	149	0.401	0.167	0.836
LO067	M	Visual	21	21	0.887	101	0.530	4.048	0.900
LO068	M	Visual	18	11	0.865	195	0.287	0.056	0.853
LO069	F	Visual	13	13	0.902	168	0.489	0.000	0.888
LO071	F	Visual	44	28	0.828	307	0.259	0.205	1.328
LO073	M	Visual	22	18	0.827	205	0.375	0.000	1.427
LO074	M	Visual	39	38	0.866	219	0.374	00.103	1.194
LO090	F	Visual	-	-	-	-	-	-	-
LO091	F	Visual	-	-	-	-	-	-	-
LY007	M	Visual	56	27	0.843	185	0.333	0.000	0.9137
LY008	M	Visual	50	18	0.775	191	0.345	0.000	1.865

Supplementary Table 1. Behavioral metrics during the visual decision-making task from the sessions of all shown in Figure 1 and Supplementary Figure 1. *LO028 and LO032 were not included in the analyses for Figure 1 because they performed the auditory version of the task and LO090 and LO091 did not pass the inclusion criteria as defined in the methods. Note that while LO037 has experienced the full set of stimuli these sessions did not pass the other inclusion criteria and were thus not included. All sessions of this subject, however, feature multiple stimulus strengths.*

Subject ID	Sex	Stimulus modality	Approximate lens coordinates in mm (AP, ML, DV)	Session date and time	Number of neurons	Presented stimulus rates	Performance on easiest trials	Number of valid trials	Early withdrawal rate	Number of no-choice trials	Trial history strength
LO028	F	Auditory	0.37, 0.5, -2	20220616_145438	464	4, 20	0.505	216	0.289	0	3.264
LO032	M	Auditory	1.21, 0.5, -1.9	20220830_121448	623	4, 20	0.375	176	0.472	0	2.058
				20220905_123313	597	4, 20	0.501	357	0.270	0	3.659
				20220907_150825	454	4, 20	0.593	378	0.200	0	2.818
				20220909_144008	527	4, 20	0.701	268	0.256	0	1.844
				20220923_135753	555	4, 6, 8, 10, 14, 16, 18, 20	0.871	342	0.240	0	1.712
LO051		Visual	1.93, 0.4, -1.9	20230322_161052	136	4, 6, 8, 16, 18, 20	0.794	242	0.226	0	1.842
				20230329_161431	224	4, 6, 8, 10, 14, 16, 18, 20	0.870	223	0.278	0	0.617
				20230427_163356	171	4, 6, 8, 10, 14, 16, 18, 20	0.900	177	0.337	1	1.512
LO067	M	Visual	1.69, 0.5, -1.7	20240209_105931	355	4, 6, 8, 10, 14, 16, 18, 20	0.849	116	0.524	13	0.510
LO068	M	Visual	1.97, 0.3, -1.5	20230831_132717	366	4, 6, 8, 10, 14, 16, 18, 20	0.861	275	0.244	0	0.782
				20230905_115256	386	4, 6, 8, 10, 14, 16, 18, 20	0.861	274	0.283	0	1.142
				20230906_140350	346	4, 6, 8, 10, 14, 16, 18, 20	0.838	201	0.290	0	1.607
				20230911_154649	414	4, 6, 8, 10, 14, 16, 18, 20	0.895	265	0.296	0	0.992
LO069	F	Visual	1.77, 0.5, -1.75	20231212_135211	306	4, 6, 8, 10, 14, 16, 18, 20	0.890	391	0.255	0	1.425
				20231227_105006	353	4, 6, 8, 10, 14, 16, 18, 20	0.926	238	0.401	0	1.099
				20240102_125134	361	4, 6, 8, 10, 14, 16, 18, 20	0.923	315	0.484	0	0.819
LO073	M	Visual	1.21, 0.8, -1.70	20240814_105301	208	4, 6, 8, 10, 14, 16, 18, 20	0.978	235	0.288	0	1.306
				20240815_110810	295	4, 6, 8, 10, 14, 16, 18, 20	0.879	404	0.260	0	2.245
LO074	M	Visual	1.69, 0.2, -1.70	20240731_165357	272	4, 6, 8, 10, 14, 16, 18, 20	0.917	192	0.430	0	1.147
				20240808_120632	195	4, 6, 8, 10, 14, 16, 18, 20	0.850	185	0.335	0	0.796

LY008	M	Visual	1.69, 0.6, -2.00	20230405_172520	*174*	*4, 6, 8, 10, 14, 16, 18, 20*	*0.880*	*184*	*0.435*	*0*	*1.316*
				20230425_105313	*125*	*4, 6, 8, 10, 14, 16, 18, 20*	*0.835*	*172*	*0.345*	*0*	*1.499*

Supplementary Table 2. Behavioral metrics, imaging coordinates and number of recorded neurons for imaging sessions included into the analyses on Figures 2, 3, and 4.
