# Supplementary material for: Anterior cingulate neurons combine outcome monitoring of past decisions with ongoing movement signals
Source: Nat Commun. 2026 Mar 24;17:4354. doi: 10.1038/s41467-026-70639-1 (PMC13171896; doi:10.1038/s41467-026-70639-1)
Supplement: Supplementary file 2 — Description of Additional Supplementary Files [file 41467_2026_70639_MOESM2_ESM.pdf]

Description of Additional Supplementary Files

File Name: Supplementary Data 1

Description: An excel spreadsheet reporting the statistical tests used and the resulting test metrics (degrees of freedom, *t*- and *F* statistics, *p*-values) grouped by figures.
